# Severe Transient Central Diabetes Insipidus After Pituitary Adenoma Removal With Peak Urine Output of 33.5 L in 24 h

**DOI:** 10.1155/crie/7516452

**Published:** 2026-05-07

**Authors:** Govinda Bhandari, Anupam Katuwal Chhetri, Rajan Gyawali, Durlab Thapa

**Affiliations:** ^1^ Department of Internal Medicine, Tribhuvan University Teaching Hospital, Kathmandu, Bagmati Province, Nepal, teachinghospital.org.np

**Keywords:** central diabetes insipidus, pituitary adenoma removal, urine output

## Abstract

**Background:**

Postoperative central diabetes insipidus (CDI) is a well‐documented but often transient complication following transsphenoidal surgery (TSS) for pituitary adenomas. CDI is characterized by excessive urine output due to impaired antidiuretic hormone (ADH) secretion following damage to the neurohypophyseal stalk or hypothalamus. While most cases resolve within a few days, severe cases with extremely high urine output are rare. This case is notable due to an exceptionally high urine output of 33.5 L in a single day, far exceeding typical postoperative presentations and highlighting the need for vigilant postoperative monitoring.

**Case Presentation:**

A 47‐year‐old female with hypertension and hypothyroidism underwent endoscopic endonasal TSS at Tribhuvan University Teaching Hospital (TUTH) for a pituitary macroadenoma compressing the optic chiasm. Within hours postoperatively, she developed significant polyuria exceeding 1000 mL/h, with urine osmolality of 213.3 mOsm/kg and hypernatremia (serum sodium 149 mmol/L), consistent with CDI. Urine output increased to 30.8 L on postoperative day 2 and peaked at 33.5 L on day 3, requiring aggressive fluid replacement and desmopressin therapy. Despite escalating doses of desmopressin, polyuria persisted before gradually resolving by postoperative day 5. Careful monitoring of electrolytes and stepwise adjustment of desmopressin led to stabilization, confirming transient rather than permanent CDI.

**Conclusion:**

This case highlights that postoperative CDI can present with extreme polyuria and rapid shifts in sodium and osmolality, making early recognition essential. It reinforces that individualized desmopressin titration and close fluid‐electrolyte monitoring are critical to prevent complications and support full recovery, especially when urine output reaches unusually high levels.

## 1. Introduction

Despite advances in transsphenoidal surgery (TSS), posterior pituitary dysfunction affecting water and electrolyte balance remains a common postoperative challenge, with central diabetes insipidus (CDI) being the most frequent disorder [1]. It can be transient either intraoperative or postoperative, which generally resolves after the third or fourth postoperative day, and can be permanent, requiring lifelong desmopressin therapy [2, 3]. In only 16% of pituitary adenomas cases, transient CDI manifests immediately after surgery and resolves within weeks, whereas refractory postoperative CDI has been reported in instances with over 85% loss of hypothalamic magnocellular neurons [2, 4]. During surgical intervention and resection of the tumor, there may be damage to the neurohypophyseal stalk or hypothalamus, leading to impaired secretion of antidiuretic hormone (ADH) [2, 5]. Factors such as larger tumor size, adherence to surrounding structures, the extent of surgical resection, and excess perioperative fluid administration can increase the likelihood of CDI development [4, 5]. Postoperative CDI is assessed by monitoring urine output, specific gravity, and serum osmolality, and is managed through careful fluid balance, administration of desmopressin, and regular electrolyte monitoring to prevent dehydration and maintain hemodynamic stability [5, 6].

Here, we present a case of a 47‐year‐old female with severe CDI with a peak urine output of 33.5 L in a day after endoscopic TSS for pituitary macroadenoma.

## 2. Case Presentation

**Figure 1:**
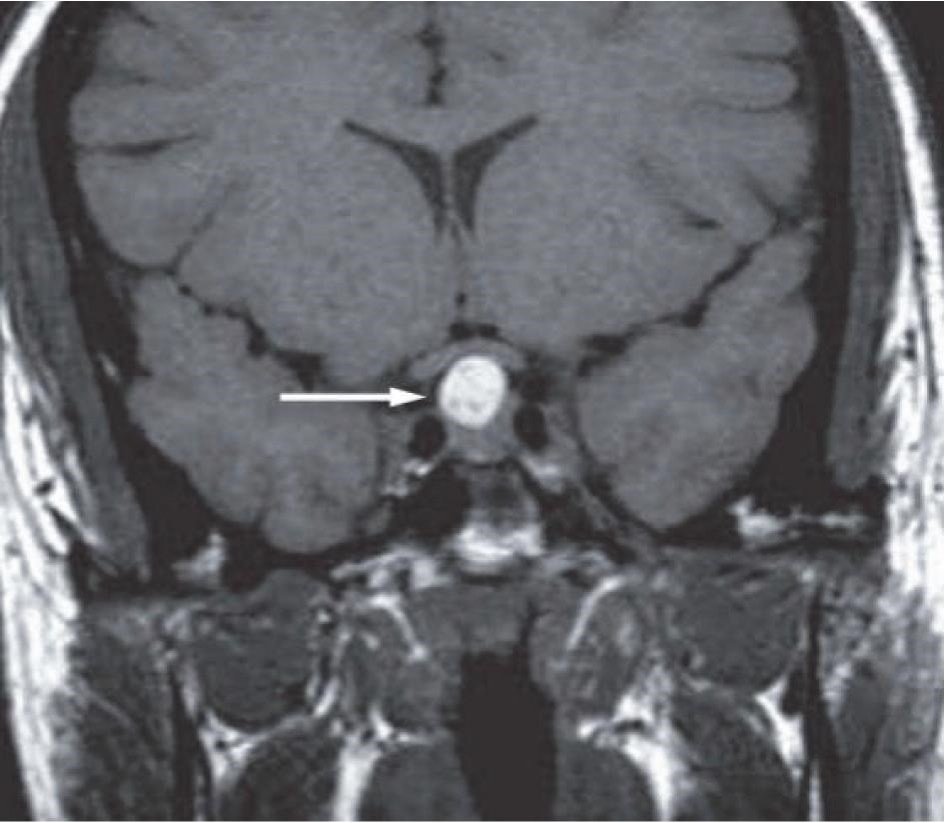
Brain MRI coronal section showing a well‐defined lobulated mass measuring 6.3 cm × 2.0 cm ×1.7 cm in the sella turcica compressing the optic chiasma.

A 47‐year‐old female with a history of hypertension for 3 years and hypothyroidism for 2 years presented with a gradual onset, progressive headache for 1 month with no diurnal variation. She also reported blurring of vision for 1 day with watering of her eyes before admission. There was no history of loss of consciousness and vomiting. Her medical history included regular medication under amlodipine and levothyroxine. On examination, MRI showed a well‐defined lobulated mass measuring 2.3 cm × 2.0 cm × 1.7 cm in sella turcica with suprasellar extension compressing optic chiasma and right cavernous sinus, suggestive of a pituitary macroadenoma (Figure 1). Preoperative hormonal levels (GH, TSH, free T4, 8 AM cortisol, FSH, and LH) were within normal limits, but prolactin was high 47.6 ng/mL (normal range 5–25 ng/mL). The elevated prolactin level is likely due to stalk effect, where compression of the pituitary stalk by the macroadenoma reduces hypothalamic dopamine inhibition, resulting in a moderate increase in prolactin. She was scheduled for an endoscopic endonasal transsphenoidal resection, which was performed by the Neurosurgery Department at Tribhuvan University Teaching Hospital (TUTH).

After surgery the patient was transferred to the postoperative ward at 5:00 PM with a Foley catheter in place for strict urine monitoring. In the immediate postoperative period, urine output was noted to be significantly elevated, reaching ~1000 mL per hour when the consultation of endocrinology was done. Over the first 12 h, total urine output was 11,800 mL, raising concerns for postoperative DI. The patient remained hemodynamically stable with a heart rate of 88 beats per minute, blood pressure of 120/75 mmHg, respiratory rate of 16 breaths per minute, and SpO_2_ of 98% on room air. Serum sodium was measured at 149 mmol/L, potassium at 3.6 mmol/L, urine specific gravity at 1.002, and urine osmolality at 213.3 mOsm/kg. These findings were consistent with CDI. The patient was started on intranasal desmopressin 10 µg once daily along with continued oral prednisolone therapy. Fluid replacement was guided by urine output, with balanced crystalloid solutions administered to maintain intravascular volume and prevent dehydration. Initially, 5% dextrose in half‐normal saline was preferred to prevent hypernatremia. The thyroid function test and cortisol level were under normal limits.

**Figure 2:**
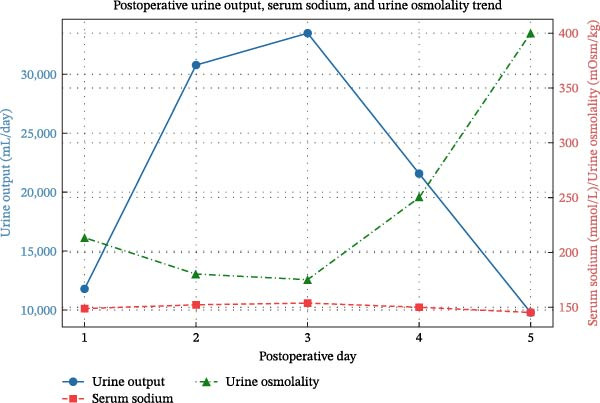
Combined graph showing the postoperative trends for urine output, serum sodium, and estimated urine osmolality.

On postoperative day 2, the urine output remained persistently high at 30,800 mL in 24 h, averaging 1500 mL per hour (Table 1). IV and oral fluids were carefully adjusted to match urine losses, and close electrolyte monitoring every 6 h was maintained. Since polyuria persisted despite conservative management, desmopressin was increased to two puffs per day. Repeat serum urea, BUN, serum creatinine, and serum potassium were within normal limits for her age On postoperative day 3, as urine output peaked at 33,500 mL in 24 h, treatment was escalated to include intravenous desmopressin 1 µg every 12 h along with oral desmopressin 0.1 mg twice daily. Blood gas analysis revealed a pH of 7.42, pCO_2_ of 44.5 mmHg, pO_2_ of 89.2 mmHg, glucose of 155 mg/dL, lactate of 3.1 mmol/L, and potassium of 3.42 mmol/L. Given the severity of polyuria and associated electrolyte imbalances, the patient’s treatment was escalated to include injectable desmopressin 1 µg along with oral desmopressin 0.1 mg twice daily. By postoperative day 4, urine output decreased to 21,600 mL per day, indicating a partial response to the combined regimen. Intravenous desmopressin was continued at 1 µg every 12 h, with ongoing oral dosing. Fluid management was continued with close monitoring of sodium and potassium levels. By postoperative day 5, urine output further declined to 9750 mL per day, suggesting stabilization of the hypothalamic–pituitary axis (Figure 2). Electrolytes remained within acceptable limits, and the patient continued oral desmopressin therapy. The gradual reduction in urine output over 5 days suggested a transient rather than permanent DI.

**Table 1:** Postoperative urine output and management.

Postoperative day	Urine output (mL/day)	Desmopressin Dose	Serum sodium (mmol/L)	Serum potassium (mmol/L)	Fluid replacement	Intervention
Day 1 (first 12 h)	11,800	Intranasal desmopressin 10 µg	149	3.6	IV ringer’s lactate, normal saline	IV fluids matched to urine loss, electrolyte monitoring
Day 2	30,800	Intranasal desmopressin 10 µg twice daily	152	3.4	IV 5% dextrose in half‐normal saline	Increased IV fluids, closer monitoring
Day 3	33,500	Inj. desmopressin 1 µg + oral 0.1 mg BD	154	3.4	IV 5% dextrose + KCl as needed	Escalated desmopressin therapy, aggressive fluid management
Day 4	21,600	Oral 0.1 mg BD	150	3.5	IV fluids tapered, oral hydration encouraged	Partial response observed, maintained IV hydration
Day 5	9750	Oral 0.1 mg BD	145	3.7	IV fluid and oral hydration	Further stabilization, tapering IV fluids

Electrolyte imbalances were managed with intravenous fluids, and by day 6, urine output stabilized, allowing a transition to oral desmopressin 0.1 mg twice daily alone. By day 7, sodium levels normalized, and the patient was discharged on oral desmopressin 0.1 mg once daily with instructions for monitoring and follow‐up. At 2 weeks, polyuria had resolved, and desmopressin was tapered. A 1‐month follow‐up confirmed stable laboratory parameters and no symptom recurrence, indicating a successful recovery.

## 3. Discussion

The central nervous system (CNS) plays a vital role in regulating water and sodium balance through a complex interaction between the hypothalamus, posterior pituitary, and adrenal glands. Disruptions in the normal function of this hypothalamus, pituitary, and adrenal axis can lead to pathological conditions such as CDI [7].

CDI is a clinical syndrome that results from absent or minimal vasopressin secretion due to damage to the hypothalamus or pituitary gland, mostly resulting from traumatic damage, as a result of traumatic brain injury or neurosurgical intervention, such as TSS, resulting in hypotonic polyuria with compensatory thirst [5]. The extreme polyuria observed in this patient likely reflects a complete transient loss of ADH, arginine vasopressin secretion, which leads to massive free water loss and hypotonic diuresis. Studies show that destruction of >90% of vasopressin‐secreting neurons is usually required to produce overt CDI [2, 5]. Additional factors, such as aggressive fluid replacement or transient hyperglycemia, can exacerbate urine output, contributing to extreme polyuria as observed in this patient [7].

Newly developed postoperative CDI or AVP‐D (arginine vasopressin deficiency) was transient in 17% of the patients, differing with tumor types: 31% of craniopharyngiomas and 16% of pituitary adenomas. Similarly, permanent AVP‐D was estimated in 3% of overall tumors; these rates differed with tumor types: pituitary adenomas 2% and craniopharyngiomas 30% [2]. Similar to this, our patient was diagnosed with a pituitary macroadenoma for which she had undergone endoscopic endonasal transsphenoidal resection.

CDI is characterized by polydipsia, polyuria, and dehydration. Diagnosis is based on the presence of hypotonic (<300 mosmol/kg), plasma sodium level (>143 mmol/L), daily urine output (>3.5 L), polyuria (>2 mL/kg/h) together with plasma osmolarity (>300 mosmol/kg), after excluding glycosuria, mannitol administration, or renal failure [8]. Postoperative cranial DI typically appears within 24 h after surgery. When the surgery is limited to the pituitary fossa, the condition is generally transient, resolving within 2–5 days. In rarer cases, cranial DI can be prolonged or permanent, often occurring after damage to the proximal pituitary stalk or following suprasellar surgeries [8]. In our case, immediately after the postoperative period, total urine output was noted to be significantly increased, raising concerns for postoperative DI. Following laboratory examinations, an increased sodium level (149 mmol/L), decreased urine osmolarity (213.3 mOsm/kg), decreased urine specific gravity (1.002), and peak urine output of 33.5 L on the 3rd postoperative day were noted and were resolving gradually at the end of the 5th postoperative day. These findings were consistent with CDI, transient in nature.

Managing CDI involves correcting the free water deficit through the proper fluid intake, replacing the missing hormone arginine vasopressin with synthetic desmopressin, which can be given through nasal, parenteral, and orally, with individualized titration of desmopressin to control symptoms while minimizing dysnatremia, addressing the underlying condition, and closely monitoring the treatment and any related causes [9]. In our case, intravenous and oral fluids were carefully adjusted to match the urine losses, desmopressin was individually titrated and given through nasal puffs, and with an increase in severity, treatment was escalated to include injectable and oral desmopressin. Urine output and electrolytes were closely monitored.

Most cases of postoperative CDI are transient, resolving within days to weeks, while permanent CDI is uncommon and usually associated with extensive pituitary or stalk injury, requiring lifelong therapy [6]. Recurrence is rare if the lesion is completely resected, but follow‐up with periodic assessment of urine output, serum sodium, and osmolality, along with patient education on polyuria and polydipsia, is essential to detect and manage any recurrence promptly [10].

## Author Contributions

Govinda Bhandari initiated and conducted the research. Govinda Bhandari and Anupam Katuwal Chhetri wrote the manuscript. Rajan Gyawali and Durlab Thapa reviewed the manuscript.

## Funding

No funding was received for this work.

## Disclosure

All authors read and approved the final manuscript.

## Ethics Statement

No ethical approval was needed.

## Consent

A written consent was given by the patient to participate in the study.

## Conflicts of Interest

The authors declare no conflicts of interest.

## Data Availability

The datasets used and/or analyzed during the current study are available from the corresponding author upon reasonable request.
